# Striatal functional connectivity alterations in mild cognitive impairment subtypes defined by CSF A/T biomarkers

**DOI:** 10.3389/fnagi.2026.1831310

**Published:** 2026-06-18

**Authors:** Yue Tang, Yiming Ruan, Darui Zheng, Yilu Huang, Wenzhang Qi, Qianqian Yuan, Chen Xue, Chaoyong Xiao

**Affiliations:** Department of Radiology, The Affliated Brain Hospital of Nanjing Medical University, Nanjing, China

**Keywords:** amyloid-β, caudate nucleus, functional connectivity, mild cognitive impairment, putamen, tau protein

## Abstract

**Background:**

Mild cognitive impairment (MCI) is the prodromal stage of Alzheimer’s disease (AD), the primary cause of dementia. In addition to supporting motor and higher-order cognitive activities, the basal ganglia, particularly the caudate nucleus and putamen, may exhibit early AD-related network changes. This study investigated differences in striatal functional connectivity (FC) across MCI subtypes marked by cerebrospinal fluid (CSF) amyloid-β (Aβ42) and phosphorylated tau (p-tau) (A/T) biomarkers (A+/A- and T+/T-), along with associations among altered FC, AD pathology, and cognitive performance.

**Methods:**

From the ADNI database, 212 individuals with MCI were stratified into three groups: A-T- (*n* = 54), A+T- (*n* = 28), and A+T+ (*n* = 52). Group differences in putamen and caudate connectivity were investigated using seed-based FC analyses. Changes in FC, CSF biomarkers, and cognitive function were evaluated using partial correlation analyses. The discriminative value of FC changes was assessed using univariate and multivariate logistic regression analyses.

**Results:**

Compared with the A-T- and A+T- groups, the A+T+ group was older and showed lower episodic memory (EM) scores. The left caudate and bilateral cerebellar anterior lobes, between the right caudate and bilateral medial frontal gyrus (MFG), and between the left putamen and left MFG all had higher FC in the A+T+ group. In the A+T+ group, right caudate–MFG connectivity was positively correlated with CSF p-tau levels (*r* = 0.424, *p* = 0.012) and negatively correlated with EM scores (*r* = -0.38, *p* = 0.018). Compared with single-region models, multivariate logistic regression demonstrated superior classification performance.

**Conclusion:**

Patients with MCI and coexisting CSF Aβ and tau pathologies (A+T+) exhibit increased FC in striatal-cortical circuits, which is strongly associated with tau pathology and episodic memory impairment. These findings provide neuroimaging evidence that striatal network alterations are linked to tau-related pathology in prodromal AD. Changes in striatal FC may serve as an early neurobiological indicator of AD-related MCI.

## 1 Introduction

Alzheimer’s disease (AD) is the leading cause of dementia and is preceded by a prodromal stage commonly referred to as mild cognitive impairment (MCI) (Velásquez et al., 2021). MCI is characterized by measurable cognitive decline that does not yet meet the diagnostic threshold for dementia (Chandra et al., 2019). However, MCI is a heterogeneous clinical condition, and not all affected individuals progress to AD. MCI can also be stratified using clinical progression patterns, such as early versus late MCI or stable versus progressive MCI, and these subgrouping approaches have also been associated with distinct functional connectivity alterations (Malotaux et al., 2023). Epidemiological studies suggest that approximately 10% of patients with MCI develop AD each year (Li et al., 2021). Approximately 15% of the population over 65 already have MCI, and after 5 years, more than half of them develop AD (Hojjati et al., 2019). Therefore, determining the underlying processes of MCI is crucial for the early detection and management of AD.

The early deposition of Aβ-amyloid (Aβ42) plaques and neurofibrillary tangles, two pathological markers specific to AD, during the MCI stage has been identified as a key predictor of disease transformation (Glodzik et al., 2011). Multiple studies have investigated the relationship between the levels of Aβ42 and tau biomarkers and the development of AD. Nevertheless, the majority of research approaches MCI as a single entity, disregarding the differences in functional compensation or brain network injury patterns that may arise from varying pathological protein levels. Clarifying the relationship between Aβ42, tau proteins, and brain function is crucial to understanding the early brain function changes in AD. This can offer valuable insights for the early diagnosis and management of MCI (Cheng et al., 2024).

Researchers have primarily focused on cortical and hippocampal changes in MCI and AD. The role of the basal ganglia, particularly the striatum (caudate nucleus and putamen), in the early AD pathophysiology remains poorly understood. Through its primary nuclei (caudate nucleus and putamen), the striatum, a key structure for motor control, cognitive processing, and emotional regulation, demonstrates important roles in neurodegenerative diseases (Serra et al., 2018). The striatum is a core component of the corticostriatal circuit, which is essential for episodic memory, executive function, and goal-directed behavior—cognitive areas that are compromised early in MCI. According to Hanseeuw et al., high Aβ42 levels in the striatum are associated with tau load levels, hippocampal atrophy, and the development of more severe cognitive impairments and dementia in the MCI stage (Hanseeuw et al., 2018). However, it is yet unknown whether striatal FC patterns vary across MCI subtypes identified by the CSF A/T framework—the gold standard for AD pathological diagnosis—and whether these FC changes are associated with Aβ or tau pathology.

By continuously collecting brain activity signals, resting-state functional magnetic resonance imaging (rs-fMRI) enables analyzing FC in specific brain regions while at rest (using seed-based analysis or independent component analysis) and elucidates multi-regional coordination patterns (using graph-based network analysis) (Storti et al., 2013). According to this technology, anomalies in the basal ganglia may dynamically evolve with disease progression, strongly correlating with the disease course and cognitive function in patients with AD (Xiong et al., 2022). Notably, rs-fMRI and task-state studies have confirmed the involvement of basal ganglia nuclei in higher-order cognitive processes through extensive FC. A significant decrease has been observed in FC between the caudate nucleus and the posterior cingulate cortex/precuneus (core hubs of the DMN) in individuals with amnesic mild cognitive impairment (aMCI) and AD, which is correlated with cognitive decline (Valera-Bermejo et al., 2021). Thus, stratified analysis of the effects of pathological proteins on functional networks in the basal ganglia can deepen the understanding of the mechanisms underlying neuronal transmission dysfunction in MCI.

Few studies have systematically investigated striatal FC changes across CSF-defined A/T biomarker subtypes in MCI. The current study aimed to elucidate whether striatal network changes are specifically related to tau pathology along the AD continuum by combining biomarker stratification with seed-based resting-state FC analysis. It was hypothesized that more substantial changes in the connection between the caudate/putamen and related brain regions occur in patients with MCI and higher levels of pathological proteins, and that these changes may be related to cognition. In addition to expanding the knowledge of the mechanisms underlying brain network compensation during the prodromal phase of AD, the findings may offer prospective biomarkers for individualized prognostic assessment, which would facilitate pathology-specific early intervention strategies.

## 2 Materials and methods

### 2.1 Participants

The Alzheimer’s Disease Neuroimaging Initiative (ADNI) database,^1^ a multicenter longitudinal research intended to validate AD-related biomarkers and monitor disease progression in MCI and early AD, provided data for this study (Sakamoto et al., 2019). The ADNI-2 operational protocols [Supplementary Information contains inclusion criteria] were followed in the enrolment of patients with MCI. These included: (1) memory complaint; (2) aberrant memory function on the Logical Memory II subscale (education-adjusted); (3) Clinical Dementia Rating (CDR) = 0.5; (4) Mini-Mental State Examination (MMSE) scores between 24 and 30; (5) neither dementia nor clinically severe depression (Geriatric Depression Scale < 6).

Initially, 226 patients with MCI having available CSF Aβ42, p-tau, and rs-fMRI data from ADNI2 and ADNI3 were included. Participants with excessive head motion during rs-fMRI scanning (cumulative translation > 3.0 mm or rotation > 3.0°) were eliminated (*n* = 14), and 212 participants were retained for final analysis. CSF Aβ42 < 977 pg/ml was defined as abnormal (A+), and CSF p-tau > 24 pg/ml was defined as abnormal (T+) (Ezzati et al., 2021; Hinrichs et al., 2011). Participants were categorized into four groups based on these criteria: A+T+ (*n* = 52), A+T- (*n* = 28), A-T+ (*n* = 78), and A-T- (*n* = 54). The A-T+ group (*n* = 78) was eliminated because its biomarker profile was inconsistent with the AD continuum under the A/T/N framework. A baseline balance test confirmed no significant differences in age, sex, and years of education between the remaining three groups (all *p* > 0.05) (Suárez-Calvet et al., 2019). Therefore, the final sample included 54 A- T-, 28 A+ T-, and 52 A+T+ patients with MCI.

### 2.2 Ethics approval and consent to participate

The ADNI trial was approved by the institutional review boards (IRBs) of all participating centers, and the experimental procedures were conducted in accordance with the tenets of the Declaration of Helsinki. Before study enrollment, each patient or their legally designated representative provided written informed consent. The official ADNI website^2^ describes the ADNI ethical approval.

### 2.3 Cognitive function

Between-group comparisons were conducted to assess cognitive functioning using composite scores for Episodic Memory (EM) and Executive Functioning (EF). Supplementary Methods contain additional information on EM and EF.

### 2.4 CSF biomarker measurement

Using the INNO-BIA AlzBio3 immunoassay kit (Fujirebio Europe N.V., Ghent, Belgium), CSF levels of amyloid-β42 (Aβ42), total tau (t-tau), and phosphorylated tau at threonine 181 (p-tau181) were quantified at the ADNI central laboratory in strict compliance with the standardized operating procedures and the ADNI biomarker testing protocol. To ensure the accuracy and repeatability of the results, each sample was processed and tested under strict quality control conditions. To improve the visualization of biomarker group differences, distributions of CSF Aβ42, p-tau181, and t-tau across the three groups were additionally displayed using boxplots (Supplementary Figure 1).

### 2.5 MRI data acquisition

At ADNI participating sites, all rs-fMRI scans were acquired using 3.0T magnetic resonance imaging (MRI) scanners (Siemens, GE Healthcare, or Philips), with scanning protocols consistent between ADNI2 and ADNI3. Detailed scan parameters and operating protocols for ADNI2 and ADNI3 can be accessed at http://adni.loni.usc.edu/wp-content/uploads/2010/05/ADNI2_MRITraining-Manual-FINAL.pdf and http://adni.loni.usc.edu/wp-content/uploads/2017/07/ADNI3-MRI-protocols.pdf.

### 2.6 Functional data preprocessing

The Data Processing and Analysis for Brain Imaging (DPABI) software^3^ within the MATLAB 2021b framework^4^ was used to preprocess the fMRI data. Preprocessing included removal of the first 10 volumes, slice-timing correction, realignment with exclusion criteria of cumulative translation/rotation > 3.0 mm/3.0°, normalization to the MNI space, spatial smoothing (6-mm FWHM), nuisance regression (24 motion parameters, WM/CSF signals, and scrubbing regressors), and temporal band-pass filtering (0.01–0.08 Hz). For additional reference, a comprehensive explanation of the procedures is provided in the Supplementary methods. No EPI distortion correction (e.g., field map- or reverse phase-encoding–based correction) was performed. Although field map data are available for a subset of participants in the ADNI dataset (e.g., ADNI3), they were not applied in the present study due to incomplete and non-harmonized availability across all subjects. Therefore, susceptibility-induced geometric distortions in frontal and temporal regions may not have been entirely corrected.

### 2.7 FC analysis

The two regions of interest (ROIs) were established based on previous research (Dong et al., 2020). Specifically, the caudate ROI was located at MNI coordinates: *X* = ± 13, *Y* = 15, *Z* = 9, whereas the putamen ROI was located at *X* = ± 28, *Y* = 1, *Z* = 3 (Peng et al., 2022). The locations of the bilateral caudate and putamen seed ROIs are illustrated in Supplementary Figure 2 to facilitate anatomical interpretation. To ensure anatomical validity of the seed regions, we performed visual inspection of ROI placement after normalization in MNI space across all participants. Particular attention was given to minimizing potential contamination from CSF or white matter signals, which may be more pronounced in aging and neurodegenerative populations. Spheres with a radius of 3 mm were used to depict these regions surrounding the central voxel at the designated coordinates. To minimize signal contamination from nearby structures and capture functionally defined subregions, spherical ROIs with a radius of 3 mm were selected (Zhang et al., 2023). The anatomical delineation of these regions has been described by Martino et al. (Di Martino et al., 2008). The sites of the two spherical ROIs did not overlap. Additionally, all functional data underwent spatial normalization and smoothing procedures, which further improved anatomical alignment across subjects. Although subject-specific manual adjustment was not performed, the consistency of ROI localization was ensured through standardized preprocessing and visual quality control. To create FC for the caudate and putamen, the averaged time course within each seed was retrieved and correlated with every voxel in the entire brain. Fisher-Z transformation was then applied to the Pearson correlation coefficients(r) between ROI activation and each voxel: *Z* = 0.5 × ln [(1 + r)/(1-r)]. Finally, four z-score maps that represented the intrinsic FC patterns were generated for each participant.

### 2.8 Statistical analysis

Statistical analyses were conducted using the Statistical Package for the Social Sciences (SPSS) software, version 22.0 (IBM, Armonk, NY, United States). The three MCI subgroups (A- T-, A+ T-, and A+T+) were compared using one-way analysis of variance (ANOVA) for continuous variables (age, years of education, neuropsychological scores, and CSF biomarker levels) and the chi-square test for categorical variables (sex). Before ANOVA analysis, the Shapiro–Wilk test and Levene’s test were used to confirm normality and homogeneity of variance. Pairwise group differences were detected using post-hoc multiple comparisons with Bonferroni correction.

A one-way ANOVA was used to evaluate FC differences in each brain region after adjusting for the confounding effects of age, sex, and years of education. Gaussian random field (GRF) correction was applied for multiple comparisons (voxel *p* < 0.005, cluster *p* < 0.05) (Zheng et al., 2025). Subsequently, the resulting masks from the ANOVA analyses were subjected to *post-hoc* pairwise comparisons using two-sample *t*-tests with the identical GRF correction criteria (voxel *p* < 0.005, cluster *p* < 0.05) (Zheng et al., 2025). Cluster-wise correction was used in accordance with standard GRF protocols to enhance robustness, and the results were interpreted cautiously. The smoothness of residuals was estimated and met the GRF assumptions.

Using age, sex, and years of education as factors, partial correlation analyses were conducted using SPSS to explore the relationship between altered FC (z-scores) in the caudate/putamen and cognitive function (EM/EF scores), as well as CSF pathological proteins (Aβ42, p-tau181). The false discovery rate (FDR) adjustment for multiple comparisons was used to modify statistical significance (*q* < 0.05).

The smoothness of residuals was computed and confirmed to meet GRF requirements to reduce the risk of inflated false-positive results associated with cluster-wise inference. Furthermore, only clusters that survived both voxel-level (*p* < 0.005) and cluster-level (*p* < 0.05, GRF corrected) thresholds were reported, and the results were interpreted cautiously. Replication in separate datasets is warranted to verify robustness owing to ongoing methodological debates about cluster-based correction.

### 2.9 Binary logistic regression analysis

Using SPSS 22.0 software, univariate and multivariate binary logistic regression were used to evaluate the diagnostic value of altered FC of each brain area in A+T- and A+T+ groups. Using the likelihood ratio and a selection criterion of *p* < 0.05, backward elimination was used to incorporate the FC changes identified through univariate analysis into the multivariable models. The receiver operating characteristic (ROC) curve and the area under the ROC curve (AUC) were used to assess the predictive performance of both univariate and multivariable models. The optimal threshold was determined using the Youden index. The results were reported in terms of accuracy, sensitivity, and specificity. In addition to distinguishing A+T+ from A- T-, a secondary analysis was performed to evaluate the discriminative ability of the multivariate model in differentiating A+T+ from A+ T-, in order to assess sensitivity to tau pathology within amyloid-positive individuals.

## 3 Results

### 3.1 Demographic and neurocognitive characteristics

The A+T+ group was older than both the A-T- and A+T- groups (Table 1). Compared with the A-T- group, the A+T+ group performed worse on the Rey Auditory Verbal Learning Test (RAVLT) in both the immediate recall and learning sections, as well as on the EM composite score. Additionally, the A+T+ group performed worse than the A+T- group on the EM score (Bonferroni-corrected, *p* < 0.05). Demographic and clinical characteristics are summarized in Table 1.

**TABLE 1:** Demographic and clinical characteristics of the participants in the A- T-, A+ T-, and A+T+ groups.

Group	A-T-	A+T-	A+T+	F-values (χ^2^)	*p*-values
Number	54	28	52		
Age (years)	68.97 (7.65)	71.68 (7.30)	73.16 (5.79)**	4.982	0.008^a^
Sex (F/M)	20/34	13/15	20/32	0.723	0.697
Years of education	15.91 (2.64)	16.46 (2.57)	16.33 (2.65)	0.534	0.588
MMSE	28.06 (1.89)	28.18 (1.74)	27.38 (2.18)	2.100	0.127
MoCA	23.79 (3.06)	23.04 (2.52)	22.76 (3.61)	1.402	0.250
RAVLT-immediate	38.43 (9.42)	34.68 (9.21)	33.10 (8.94)*	4.620	0.012^a^
RAVLT-learning	4.91 (2.24)	4.36 (2.09)	3.73 (2.39)*	3.559	0.031^a^
RAVLT-forgetting	4.43 (3.93)	4.57 (2.17)	5.10 (2.51)	0.654	0.522
RAVLT-prec-forgetting	46.08 (56.25)	57.15 (29.42)	65.50 (30.51)	2.757	0.067
LDELTOTAL	8.20 (2.54)	6.21 (3.06)*	5.90 (3.34)***	8.809	< 0.001^ab^
EM	0.49 (1.20)	0.30 (1.05)	0.05 (0.65)**	5.113	0.007^a^
EF	0.65 (0.91)	0.23 (1.05)	0.24 (0.84)	3.310	0.040
Aβ_42_	1484.25 (255.03)	726.19 (199.64)***	622.09 (159.11)***	249.840	< 0.001^ab^
T-tau	201.89 (44.35)	178.88 (46.61)	383.71 (130.72)***/***	73.112	< 0.001^ac^
P-tau	17.36 (3.98)	16.30 (4.68)	40.58 (16.54)***/***	75.415	< 0.001^ac^

Data are presented as mean (standard deviation) unless otherwise indicated. Group differences were assessed using one-way ANOVA for continuous variables and chi-square tests for categorical variables.

*p < 0.05, **p < 0.01, ***p < 0.001 (Bonferroni-corrected for multiple comparisons). MMSE, Mini-Mental State Examination; MoCA, Montreal Cognitive Assessment; RAVLT, Rey Auditory Verbal Learning Test; LDELTOTAL, Logical Memory Delayed Recall Total Score; EM, Episodic Memory; EF, Executive Function; Aβ42, amyloid-beta 42; t-tau, total tau; p-tau, phosphorylated tau.

^a^*Post-hoc* analyses showed a significantly group difference between A+T+and A-T-.

^b^*Post hoc* analyses showed a significantly group difference between A+T- and A-T-.

^c^*Post-hoc* analyses showed a significantly group difference between A+T+ and A+T-.

### 3.2 Caudate connectivity and putamen connectivity

**FIGURE 1:**
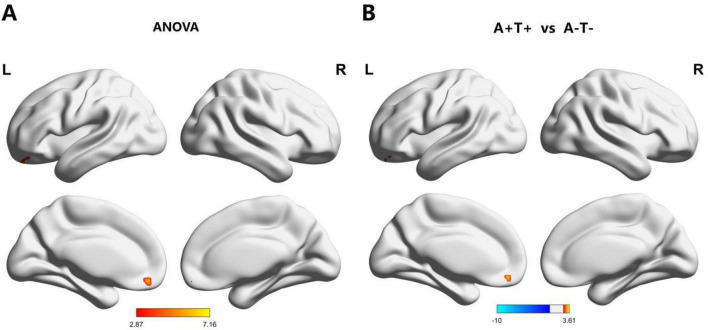
Brain regions showing significant between-group differences in FC with the left caudate nucleus. **(A)** Brain regions showing significant group differences in FC with the left caudate nucleus across the A- T-, A+ T-, and A+T+ groups, identified by one-way ANOVA (GRF-corrected, voxel *p* < 0.005, cluster *p* < 0.05). **(B)** *Post-hoc* comparison showing regions with significantly increased left caudate connectivity in the A+T+ group relative to the A-T- group. Color bars indicate statistical values (*F*-values in panel A; *t*-values in **B**). A+T+ denotes abnormal Aβ42 and p-tau; A+T- denotes abnormal Aβ42 with normal p-tau; A-T- denotes normal Aβ42 and p-tau.

ANOVA analysis showed that three groups, including bilateral cerebellar posterior lobe (CPL) and cerebellar anterior lobe (CAL), had significantly different FC in the left caudate nucleus network (Figure 1 and Table 2). Compared with the A-T- group, the A+T+ group exhibited higher FC in bilateral CAL (GRF corrected, voxel *p* < 0.005, and cluster *p* < 0.05). These analyses were adjusted for age, sex, and years of education.

**TABLE 2:** Differences in FC of the left caudate nucleus across three groups.

Region	Peak MNI coordinate			*F/t*	Cluster number
	X (MNI)	Y (MNI)	Z (MNI)		
ANOVA					
R cerebellum posterior lobe	3	–48	–39	7.7293	102
B cerebellum posterior lobe	12	–60	–54	6.3373	106
B cerebellum anterior lobe	6	–21	–27	8.301	475
A+T+ vs. A-T-					
B cerebellum anterior lobe	–3	–30	–27	3.632	432

X, Y, and Z coordinates indicate the peak MNI locations. Cluster size > 10 voxels in the ANOVA analysis, GRF corrected, voxel *p* < 0.005, cluster *p* < 0.05; Cluster size > 10 voxels in the *post-hoc* test, GRF corrected, voxel *p* < 0.005, cluster p < 0.05; A+T+, abnormal Aβ42 and p-tau; A+ T-, abnormal Aβ42 and normal p-tau; A- T-, normal Aβ42 and p-tau; B, bilateral; L, left; and R, right.

**FIGURE 2:**
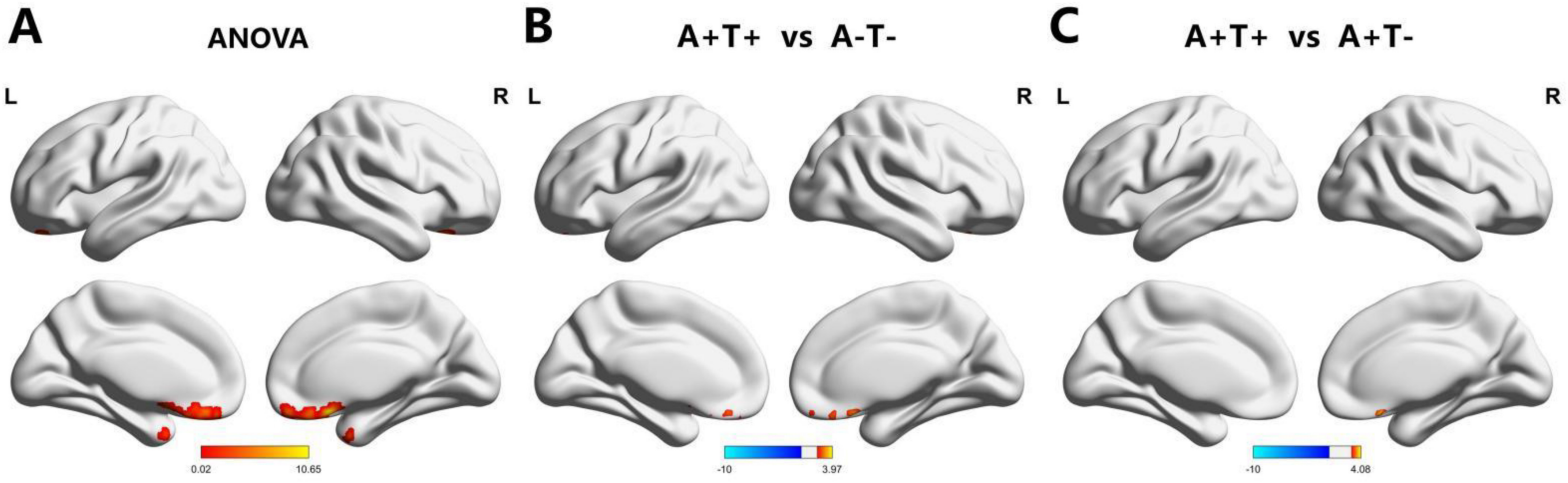
Brain regions showing significant between-group differences in FC with the right caudate nucleus. **(A)** Brain regions showing significant group differences in FC with the right caudate nucleus among the A- T-, A+ T-, and A+T+ groups, identified by one-way ANOVA (GRF-corrected, voxel *p* < 0.005, cluster *p* < 0.05). **(B)** *Post-hoc* comparison showing regions with significantly increased right caudate connectivity in the A+T+ group relative to the A-T- group. **(C)** *Post-hoc* comparison showing regions with significantly increased right caudate connectivity in the A+T+ group relative to the A+T- group. Color bars indicate statistical values (F values in panel A; t values in **(B,C)**. A+T+ denotes abnormal Aβ42 and p-tau; A+T- denotes abnormal Aβ42 with normal p-tau; A-T- denotes normal Aβ42 and p-tau.

**FIGURE 3:**
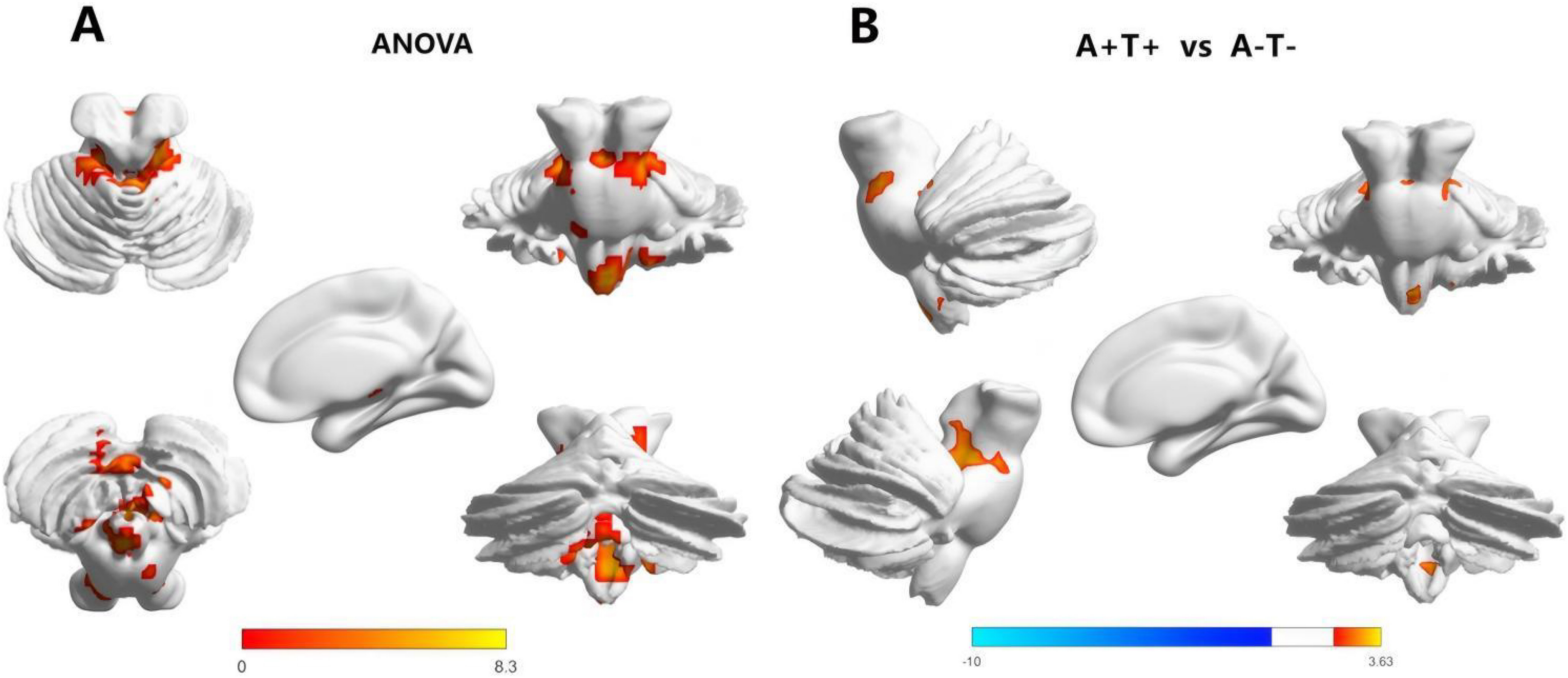
Brain regions showing significant between-group differences in FC with the left putamen. **(A)** Brain regions showing significant group differences in FC with the left putamen across the A- T-, A+ T-, and A+T+ groups, identified by one-way ANOVA (GRF-corrected, voxel *p* < 0.005, cluster *p* < 0.05). **(B)** *Post-hoc* comparison showing regions with significantly increased left putamen connectivity in the A+T+ group relative to the A-T- group. Color bars indicate statistical values (F values in panel A; t values in panel B). A+T+ denotes abnormal Aβ42 and p-tau; A+T- denotes abnormal Aβ42 with normal p-tau; A-T- denotes normal Aβ42 and p-tau.

ANOVA showed significant differences in FC among the three groups, including the bilateral medial frontal gyrus (MFG), in the right caudate nucleus cortex network. Significant differences in the right caudate network are shown in Figure 2 and Table 3. Compared with the A-T- group, the A+T+ group demonstrated higher bilateral MFG connectivity. Compared with the A+T- group, the A+T+ group showed higher FC in the right MFG (GRF corrected, voxel *p* < 0.005, and cluster *p* < 0.05). These results were adjusted for age, sex, and years of education. ANOVA analysis showed significant changes in FC among the three groups, including the left MFG, in the left putamen-cortical network. Altered FC in the left putamen network is summarized in Figure 3 and Table 4. Compared with the A-T- group, the A++T++ group showed a higher FC in the left MFG (GRF corrected, voxel *p* < 0.005, and cluster *p* < 0.05). These results were adjusted for age, sex, and years of education.

**TABLE 3:** Differences in FC of the right caudate nucleus across three groups.

Region	Peak MNI coordinate			*F/t*	Cluster number
	X (MNI)	Y (MNI)	Z (MNI)		
ANOVA					
B medial frontal gyrus	3	21	–18	10.6483	378
A+T+ VS. A-T-					
B medial frontal gyrus	–9	45	–30	3.9697	322
A+T+ vs. A+T-					
R medial frontal gyrus	3	21	–18	4.0759	22

X, Y, and Z coordinates indicate the peak MNI locations. Cluster size > 10 voxels in the ANOVA analysis, GRF corrected, voxel *p* < 0.005, cluster *p* < 0.05; Cluster size > 10 voxels in the *post-hoc* test, GRF corrected, voxel *p* < 0.005, cluster *p* < 0.05; A+T+, abnormal Aβ42 and p-tau; A+ T-, abnormal Aβ42 and normal p-tau; A- T-, normal Aβ42 and p-tau; B, bilateral; L, left; and R, right.

**TABLE 4:** Differences in FC of the left putamen across three groups.

Region	Peak MNI coordinate			*F/t*	Cluster number
	X (MNI)	Y (MNI)	Z (MNI)		
ANOVA					
L medial frontal gyrus	-24	45	-21	7.162	106
A+T+ vs. A-T-					
L medial frontal gyrus	-24	45	-21	3.6107	86

X, Y, and Z coordinates indicate the peak MNI locations. Cluster size > 10 voxels in the ANOVA analysis, GRF corrected, voxel *p* < 0.005, cluster *p* < 0.05; Cluster size > 10 voxels in the *post-hoc* test, GRF corrected, voxel *p* < 0.005, cluster *p* < 0.05; A+T+, abnormal Aβ42 and p-tau; A+ T-, abnormal Aβ42 and normal p-tau; A- T-, normal Aβ42 and p-tau; B, bilateral; L, left; and R, right.

### 3.3 Correlation analysis

**FIGURE 4:**
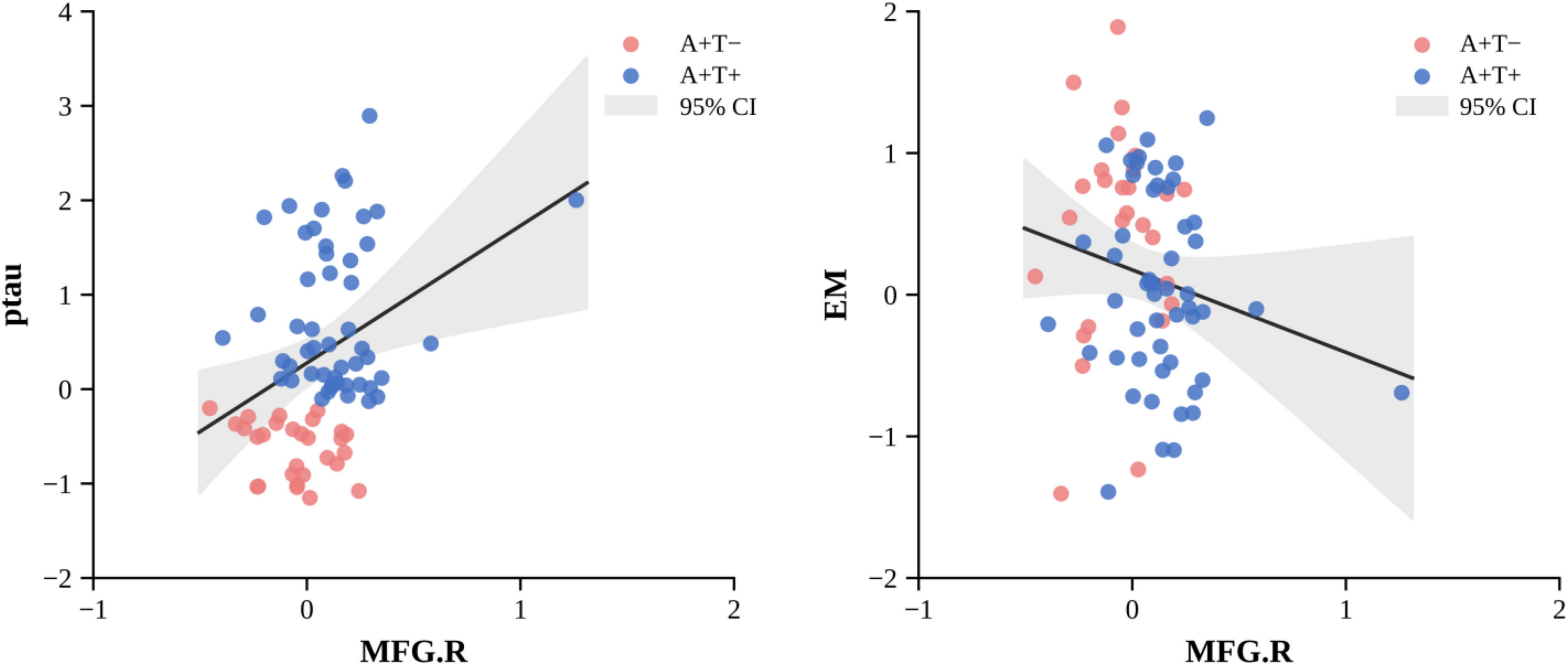
Association between FC changes in the right caudate nucleus and cognitive function. Covariates include age, sex, and years of education. Results remained significant after exclusion of the potential influential point. A+T+ denotes abnormal Aβ42 and p-tau; A+T- denotes abnormal Aβ42 with normal p-tau.

Partial correlation results are illustrated in Figure 4. In the A+T+ group, the FC of the right MFG was positively correlated with CSF p-tau (*r* = 0.424, *p* = 0.012), but negatively correlated with the EM score (*r* = -0.38, *p* = 0.018). Moreover, it withstood FDR correction (*q* < 0.05). To assess the robustness of the observed association, a sensitivity analysis was conducted by excluding the potential influential data point. The relationship between FC and CSF p-tau (and EM score) remained significant (*p* < 0.05), indicating that the findings were not driven by a single observation.

### 3.4 ROC curve

**FIGURE 5:**
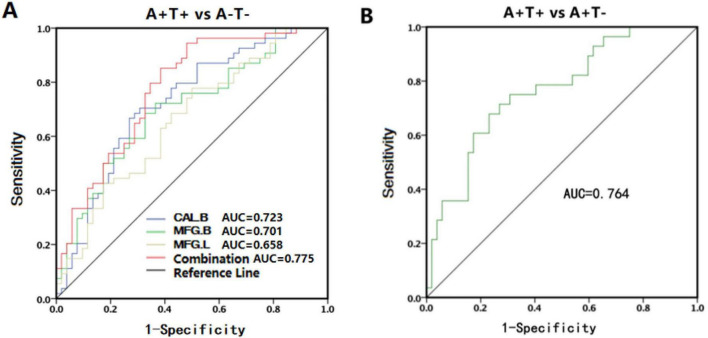
Receiver operating characteristic curves showing the classification performance of individual FC features and the multivariable logistic regression model. **(A)** ROC curves for differentiating A+T+ from A-T- MCI subtypes; **(B)** ROC curves for differentiating A+T+ from A+T- MCI subtypes. The multivariable model incorporated altered FC measures that were significant in univariate analyses and was adjusted for age, sex, and years of education. The area under the curve (AUC), sensitivity, and specificity are reported in the Results section. Each ROC curve is labeled with the corresponding feature, and AUC values are displayed within the panel for clarity. FC, functional connectivity; MCI, mild cognitive impairment.

Figure 5 illustrates the ROC curves for each change. With an AUC value of 0.723 (95% CI: 0.625–0.820, *p* < 0.001), the FC of the bilateral CAL showed good classification efficiency for differentiating A+T+ from A-T-. At the optimal cutoff determined by the Youden index, the sensitivity and specificity were 73.1 and 66.7%, respectively. However, the multivariate model (red line), which incorporated the FC changes in several different brain regions, was the best-fitting model. After adjusting for age, sex, and educational level, the AUC value increased to 0.775 (95% CI: 0.686–0.863, p < 0.001), with sensitivity of 61.5% and specificity of 83.2%. In addition, the FC between the right caudate and MFG demonstrated moderate classification performance, with an AUC of 0.764 (95% CI: 0.657–0.872, p < 0.001), indicating its potential sensitivity to tau-related pathological differences within amyloid-positive MCI.

## 4 Discussion

This study investigated striatal FC changes across CSF A/T- defined MCI subtypes. Compared with the A-T- group, patients in the A+T+ group exhibited significantly higher FC within striatal–cortical circuits, including bilateral caudate–cerebellar anterior lobe connectivity, bilateral caudate–medial frontal gyrus (MFG) connectivity, and left putamen–MFG connectivity. Importantly, higher FC between the right caudate and MFG was positively associated with CSF p-tau levels and negatively associated with episodic memory performance.

Beyond their classical role in motor control, the basal ganglia contribute substantially to executive control, reinforcement learning, action selection, and memory-guided behavior through extensive cortico-striato-thalamo-cortical loops (Simonyan, 2019) . In the context of MCI and AD, disruption of these circuits may impair the integration of cognitive control and memory-related processes, thereby contributing to executive dysfunction and episodic memory decline (Liu et al., 2023; O’Callaghan et al., 2014). Increasing evidence indicates that striatal abnormalities, including hypometabolism, atrophy, and disrupted connectivity with prefrontal regions, are associated with executive deficits and broader cognitive decline in AD (Liu et al., 2023). Our findings support the view that striatal network abnormalities are not merely secondary changes, but may represent a relevant component of early AD-related network dysfunction.

Therefore, rather than isolated amyloid accumulation, striatal hyperconnectivity is specifically related to the coexistence of amyloid and tau pathology. The positive correlation with p-tau levels indicates that altered corticostriatal signaling and enhanced local neuronal excitability may be associated with tau-related synaptic dysfunction. Meanwhile, a merely compensatory view is refuted by the negative association with episodic memory performance. Alternatively, the observed hyperconnectivity may be attributed to maladaptive network remodeling or early network instability during the prodromal stage of AD.

Increased connectivity has been observed in most studies investigating FC changes associated with tau deposition. Despite appearing to be contradictory to the typical pattern of functional decline in neurodegenerative diseases, the reported phenomena of FC augmentation are consistent with related studies on early neuroplasticity and compensatory mechanisms within the AD spectrum. The processing demands on brain networks rise with tau accumulation. Hyperconnectivity within this network may result from compensatory shifts toward the pDMN (Hasani et al., 2021). According to Pasquini et al., FC within the pDMN is positively correlated with levels of Aβ42 and tau protein deposits throughout the cerebral cortex. Specifically, raised pathological protein deposition correlates with stronger pDMN FC (Ai et al., 2025). This study provides new evidence: early MCI may cause hyperactivation of basal ganglia regions—particularly the caudate and putamen—which are core components of the prefrontal-striatal cognitive circuit. Enhanced FC may reflect an early network rearrangement process. Such hyperconnectivity may indicate a transient compensatory response to pathological burden as well as impending network instability (Zamani et al., 2025). Determining whether these changes are adaptive or maladaptive is still challenging.

However, this “enhancement” can be dual in nature. In the A+T+ group, the MFG connection strength was positively correlated with p-tau but negatively correlated with EM scores. This finding suggests that hyperconnectivity may reflect network degradation or impairment attributable to tau pathology. Reduced information-processing efficiency is one possible explanation: as the disease exacerbates, neural circuits require more resources to perform the same tasks, which eventually results in cognitive impairment. Furthermore, this result is consistent with the A+T+ group’s worse performance on the RAVLT’s immediate recall and learning subtests. Striatal–cortical hyperconnectivity may represent a dynamic process that changes from adaptive reorganization to maladaptive network inefficiency with pathology progression. However, this hypothesis warrants longitudinal validation.

Additionally, compared with the A-T- group, the A+T- group did not show significant FC differences between the caudate and putamen and other brain regions. Thus, isolated Aβ pathology may be insufficient to disrupt functional networks in early MCI. This finding is in line with the “threshold model” of AD proposed by Jack et al. According to this model, Aβ deposition triggers initial synaptic toxicity; nevertheless, synergistic tau pathology is necessary to break the threshold for neural network compensation (Jack et al., 2018). In this model, extensive neurological impairment requires the synergistic accumulation of Aβ and tau proteins. Aβ alone may cause synaptic toxicity; however, it has a marginal effect on large-scale networks in the absence of tau protein propagation (Xie et al., 2025). This is consistent with the clinical heterogeneity of MCI, suggesting that dementia usually progresses more slowly in Aβ-positive individuals without tauopathy. These findings are specific to the AD continuum, as evidenced by the presence of an A-T+ group that was excluded because of non-AD pathology. However, this exclusion raises questions about the function of non-AD tauopathies in MCI and necessitates more research into different pathways.

Finally, ROC analysis demonstrated that FC changes may serve as a neurobiological marker that differentiates A+T+ from A-T-. Specifically, the FC of the bilateral CAL alone yielded a significant AUC of 0.723, indicating a passably strong discriminatory ability. This finding challenges the conventional theory that only considers cortical and hippocampal regions by associating cerebellar circuits with the early pathophysiological processes of AD.

Moreover, compared with single-region models, the multivariable model showed higher AUC values. However, these findings should be interpreted cautiously owing to the small sample size and lack of external validation. The classification results primarily suggest that combining several striatal FC characteristics may enhance discriminatory performance, warranting more research in larger independent cohorts.

## 5 Limitations

Several limitations should be acknowledged. First, causal inferences about Aβ/tau dynamics and FC changes were impossible because of the cross-sectional research design. Longitudinal studies are warranted to investigate how these relationships evolve with disease progression, particularly during the transition from MCI to AD dementia. Second, although reliable, the use of CSF biomarkers failed to capture regional patterns of protein deposition that may influence FC heterogeneity. Future studies incorporating PET imaging for Aβ42 and tau may refine spatial correlations between AD pathology and network disruptions. Third, the small sample size, particularly in the A+T- group (*n* = 28), may limit the statistical power to identify subtle group differences. Larger cohorts with balanced subgroup sizes are warranted to validate these findings. Moreover, there is a likelihood of inflated false positives upon using GRF correction with a voxel-level threshold of *p* < 0.005. Therefore, replication in independent cohorts is warranted. Furthermore, susceptibility-related EPI distortions were not explicitly corrected in the present analysis. Although such correction was not available for all included datasets in a harmonized manner, we acknowledge that ADNI3 includes field map acquisitions that may enable susceptibility distortion correction in future analyses. Another limitation of this study is the absence of structural covariates (e.g., intracranial volume or regional gray matter volume) in the statistical models. Given that MCI populations are characterized by varying degrees of brain atrophy, partial volume effects may have influenced the estimation of functional connectivity. Although nuisance regression (including WM/CSF signals) was applied to reduce non-neuronal noise, these steps do not fully account for underlying structural variability. Future studies integrating structural MRI measures, such as voxel-wise gray matter masking or volumetric covariates, are warranted to better disentangle functional alterations from structural changes. Finally, the ROI-based approach focused on the caudate and putamen; adding graph theory measures or expanding analyses to whole-brain networks may uncover additional network-level anomalies.

## 6 Conclusion

To conclude, the co-pathology of Aβ42 and tau in MCI is associated with distinct FC changes in the caudate-putamen networks, which are correlated with cognitive impairment. These results emphasize the potential of FC measures as early markers of AD-related network disruption. Additionally, striatal FC changes may serve as an early neurobiological marker for AD-related MCI. Future longitudinal and multi-modal imaging studies will confirm the clinical value of striatal FC abnormalities in early diagnosis of AD and its progression prediction.

## Data Availability

Publicly available datasets were analyzed in this study. This data can be found here: http://adni.loni.usc.edu/wp-content/uploads/how_to_apply/ADNI_Acknowledgement_List.pdf.

## Footnotes

1
http://adni.loni.usc.edu

2
www.adni-info.org

3
http://rfmri.org/DPABI

4
http://www.mathworks.com/products/matlab/
