# Supplementary material for: Striatal functional connectivity alterations in mild cognitive impairment subtypes defined by CSF A/T biomarkers
Source: Front Aging Neurosci. 2026 Jun 18;18:1831310. doi: 10.3389/fnagi.2026.1831310 (PMC13323011; doi:10.3389/fnagi.2026.1831310)
Supplement: Supplementary file 1 [file Table_1.docx]

**Materials and methods**

**Participants**

The inclusion of MCI patients was based on the ADNI-2 procedures manual: 1) memory complaint; 2) Abnormal memory function documented by scoring within the education adjusted ranges on the Logical Memory II subscale (Delayed Paragraph Recall, Paragraph A only) from the Wechsler Memory Scale Revised (≤ 8 for 16 or more years of education; ≤ 4 for 8-15 years of education; ≤ 2 for 0-7 years of education); 3) Clinical Dementia Rating (CDR) = 0.5; 4) Mini-Mental State Examination (MMSE) scores between 24 and 30; 5) no dementia and no evidence of clinically significant depression (GDS < 6). The composite scores were derived from ADNI-provided standardized z-scores.

**Neuropsychological Assessment**

For assessing cognitive function, we conducted comparisons between groups using the composite episodic memory (EM) score and the composite executive function (EF) score. The composite EM score consisted of the Rey Auditory Verbal Learning Test (RAVLT), the word list learning and recognition components of the Alzheimer’s Disease Assessment Scale–Cognitive (ADAS-Cog), the word recall items of the Mini-Mental State Examination (MMSE), and Logical Memory I from the Wechsler Memory Scale–Revised (WMS-R). The composite EF score comprised the digit symbol substitution and digit span backward tests, trail making test parts A and B, animal and vegetable category fluency, digit cancellation, and the clock drawing test.

**Functional data preprocessing**

The preprocessing of fMRI data was conducted using the Data Processing and Analysis for Brain Imaging (DPABI) software (http://rfmri.org/DPABI) within the MATLAB 2021b framework (<http://www.mathworks.com/products/matlab/>).

The process began by discarding the first 10 volumes to enhance the stability of the MRI signal. Subsequently, slice timing correction and head movement correction were applied. Subjects' images were excluded if the translation or rotation exceeded 3 mm or 3°, respectively. The images were then spatially normalized to the MNI echo-planar imaging template and resampled to a default voxel size of 3 x 3 x 3 mm³. To minimize confounding effects on the dependent variable, nuisance covariates—such as 24 motion parameters, white matter signal, and cerebrospinal fluid signal—were regressed out. Remove the global signal to enhance signal quality. High-frequency noise was reduced by applying a 6 x 6 x 6 mm full-width at half maximum (FWHM) Gaussian smoothing filter. Finally, a temporal bandpass filter (0.01–0.08 Hz) was applied to eliminate low-frequency drifts and high-frequency noise.
