# Supplementary material for: Striatal functional connectivity alterations in mild cognitive impairment subtypes defined by CSF A/T biomarkers
Source: Front Aging Neurosci. 2026 Jun 18;18:1831310. doi: 10.3389/fnagi.2026.1831310 (PMC13323011; doi:10.3389/fnagi.2026.1831310)
Supplement: Supplementary file 2 [file Data_Sheet_1.docx]

Supplementary Figure S1


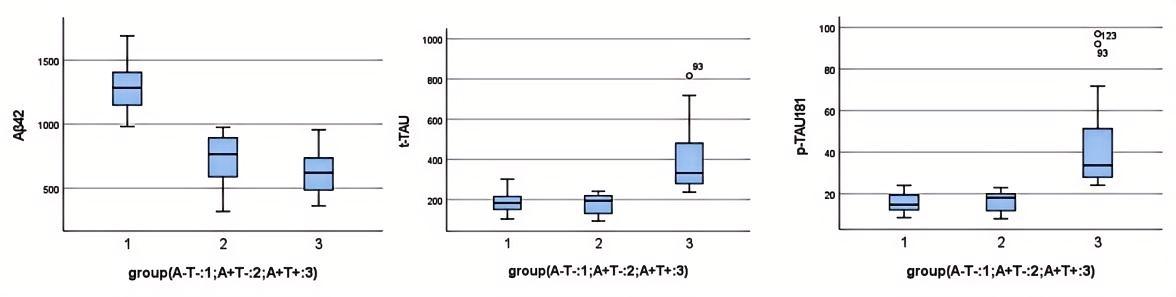


Fig.S1. Boxplots of CSF biomarkers across groups.

Boxplots show the distribution, variability, and potential outliers of (A) Aβ42, (B) t-tau, and (C) p-tau181 in A−T−, A+T−, and A+T+ subgroups.

Supplementary Figure S2


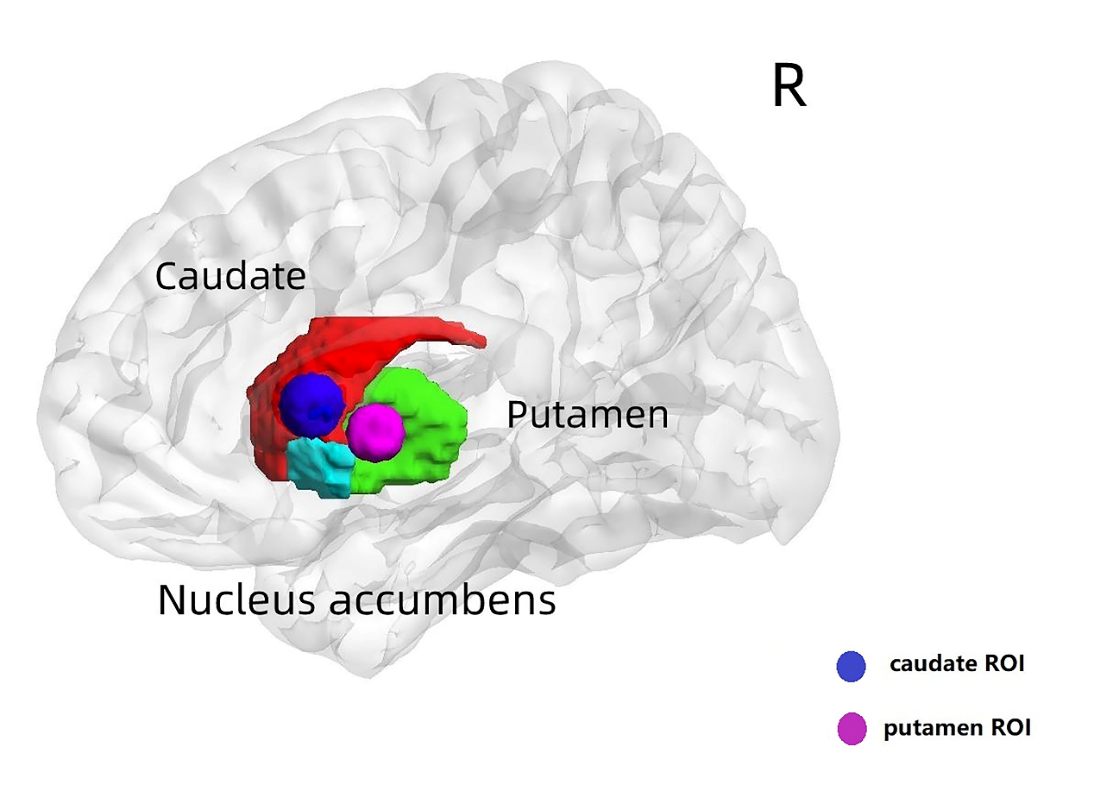


Fig. S2. The location of two spherical ROIs.

This figure illustrates the location of the two spherical ROIs, demonstrating that there is no spatial overlap between them.
